# Fungal Malignant Otitis Externa: A Systematic Review

**DOI:** 10.7759/cureus.71345

**Published:** 2024-10-13

**Authors:** Giorgos Sideris, Dioni-Pinelopi Petsiou, Melina Kourklidou, Nikolaos Papadimitriou, Petros V Vlastarakos, Sotirios Karamagkiolas, Ioannis Margaris, Thomas Nikolopoulos, Alexander Delides

**Affiliations:** 1 2nd ENT Department, Attikon University Hospital, National and Kapodistrian University of Athens, Athens, GRC; 2 4th Department of Surgery, Attikon University Hospital, National and Kapodistrian University of Athens, Athens, GRC

**Keywords:** fungal malignant otitis externa, fungi, malignant otitis externa, necrotising otitis externa, skull base osteomyelitis

## Abstract

Malignant otitis externa (MOE) is a rare but serious condition primarily caused by *Pseudomonas aeruginosa*. Fungi, particularly in immunocompromised individuals, can also be a contributing factor. A systematic review was conducted, following PRISMA guidelines, to evaluate the literature on fungal malignant otitis externa (FMOE), focusing on its etiology, patient demographics, clinical presentation, and treatment. Out of 464 articles identified, 10 were analyzed in detail, involving 197 patients with a mean age of 65.9 years. Of the total patients, 143 were male (72.6%), 52 were female (26.4%), and the gender of the remaining two was not specified. One hundred and fifty-five patients (78.7%) had underlying immunosuppressive conditions such as diabetes mellitus, chronic kidney failure, corticosteroid use, chemotherapy, and AIDS. Fungal cultures were positive in 107 cases (54.3%), with *Candida* and *Aspergillus* species being present in nearly equal proportions. There were no significant differences between fungal and non-fungal MOE in terms of clinical presentation and diagnostic methods. Conservative treatment was used in 179 patients (90.8%), with 172 of them (87.3%) receiving antifungals. Itraconazole and voriconazole were the most common antifungals, while amphotericin B was less frequently used due to side effects. In some cases, antifungals were combined with antibiotics. Surgical interventions were performed in 35 patients (17.8%), and hyperbaric oxygen therapy was used in 34 of them (17.3%). Eight patients with FMOE died, and the mortality rate was 4%. Late diagnosis, cranial nerve involvement, and inadequate treatment may contribute to higher mortality. Given the potential underdiagnosis of FMOE, early incorporation of antifungal medications into empirical treatment protocols could improve outcomes for patients with a poor prognosis.

## Introduction and background

Malignant otitis externa (MOE), also referred to as necrotizing otitis externa (NOE), is a relatively rare but severe condition originating in the external auditory canal. It can be caused by a variety of pathogens, with *Pseudomonas aeruginosa* responsible for up to 90% of cases. Other causative organisms include *Staphylococcus* species, *Klebsiella*, *Proteus*, and various fungi [1-4]. Regardless of the pathogen, MOE can lead to life-threatening complications such as skull base osteomyelitis, sigmoid sinus thrombosis, meningitis, and sepsis if left untreated [2].

Although Chandler described the first series of MOE patients in 1968, it was Cohen and Friedman’s seminal paper in 1987 that established the major and minor criteria for diagnosing MOE [5-6]. Major criteria include pain, edema, exudate, granulation tissue, micro-abscesses (in surgical cases), a positive technetium-99 (99Tc) bone scan, and failure of treatment after a week of topical therapy. Minor criteria consist of positive cultures for *Pseudomonas* from the external auditory canal, diabetes mellitus (DM), cranial neuropathies, positive CT scan, debilitating conditions, and advanced age. According to the authors, all major criteria must be present for a definitive diagnosis of MOE [6]. The presence of only minor criteria is insufficient to confirm the diagnosis, indicating that MOE does not necessarily require the isolation of *Pseudomonas aeruginosa* in cultures for diagnosis.

The first case of fungal involvement in MOE was reported in 1985 [7]. Fungi are rarely associated with MOE and are primarily observed in immunocompromised patients with conditions such as human immunodeficiency virus (HIV) infection or acute leukemia. Aspergillus fumigatus is the most common pathogen involved in fungal MOE (FMOE), particularly in patients with severe neutropenia due to HIV infection, leukemia, chronic corticosteroid use, or other causes [8]. FMOE carries high morbidity and mortality rates, often due to delayed diagnosis and underlying patient comorbidities. Additionally, systemic toxicity from common antifungal medications may result in suboptimal treatment, contributing to the disease’s poor outcomes [8].

The primary treatment for MOE, whether fungal or non-fungal, involves long-term intravenous antibiotics (e.g., aminoglycosides, antipseudomonal β-lactam agents, fluoroquinolones), guided by culture results and antibiograms [9]. In cases of FMOE, antifungal agents such as amphotericin B, voriconazole, or itraconazole are used alongside antibiotics [10]. Optional supportive treatments include surgical debridement and hyperbaric oxygen therapy, although evidence supporting the latter’s efficacy is limited [11].

Due to the rarity of FMOE, there are few original studies on this topic. However, its complex nature and high mortality rates highlight the importance of further investigation into this challenging disease. This systematic review aims to provide a comprehensive overview of the available data regarding the etiology, patient characteristics, clinical manifestations, treatment protocols, and outcomes of patients with FMOE.

## Review

Materials and methods

This systematic review followed PRISMA guidelines for systematic reviews [12]. A literature search was conducted by three authors in PubMed and Cochrane databases, with the final search on December 31, 2023. The search terms used were: (malignant OR necrotizing) AND (otitis externa) AND (fungus OR *Aspergillus* OR *Candida*), as well as (necrotizing) NEXT otitis NEXT externa OR malignant NEXT otitis NEXT externa OR otitis NEXT externa NEXT (fungal OR *Aspergillus* OR *Candida*).

Inclusion criteria were original research articles on fungal MOE, including randomized trials and observational studies. Exclusion criteria included non-English texts, reviews, case reports with fewer than five patients, and animal studies.

The search strategy initially identified 464 articles. After removing one duplicate, 463 unique records were screened based on abstract availability, relevance to the topic, and language of publication. A total of 151 articles were excluded as off-topic, 72 lacked available abstracts, and 31 were written in languages other than English. This left 209 reports for further retrieval. After applying the exclusion criteria, 142 records were excluded: 44 review articles, 84 case reports, seven case series with fewer than five patients, one editorial, and six animal studies. Full-text screening and eligibility evaluation of the remaining 67 articles led to the exclusion of 57, leaving 10 studies that met all the eligibility criteria and were included in this review.

**Figure 1:**
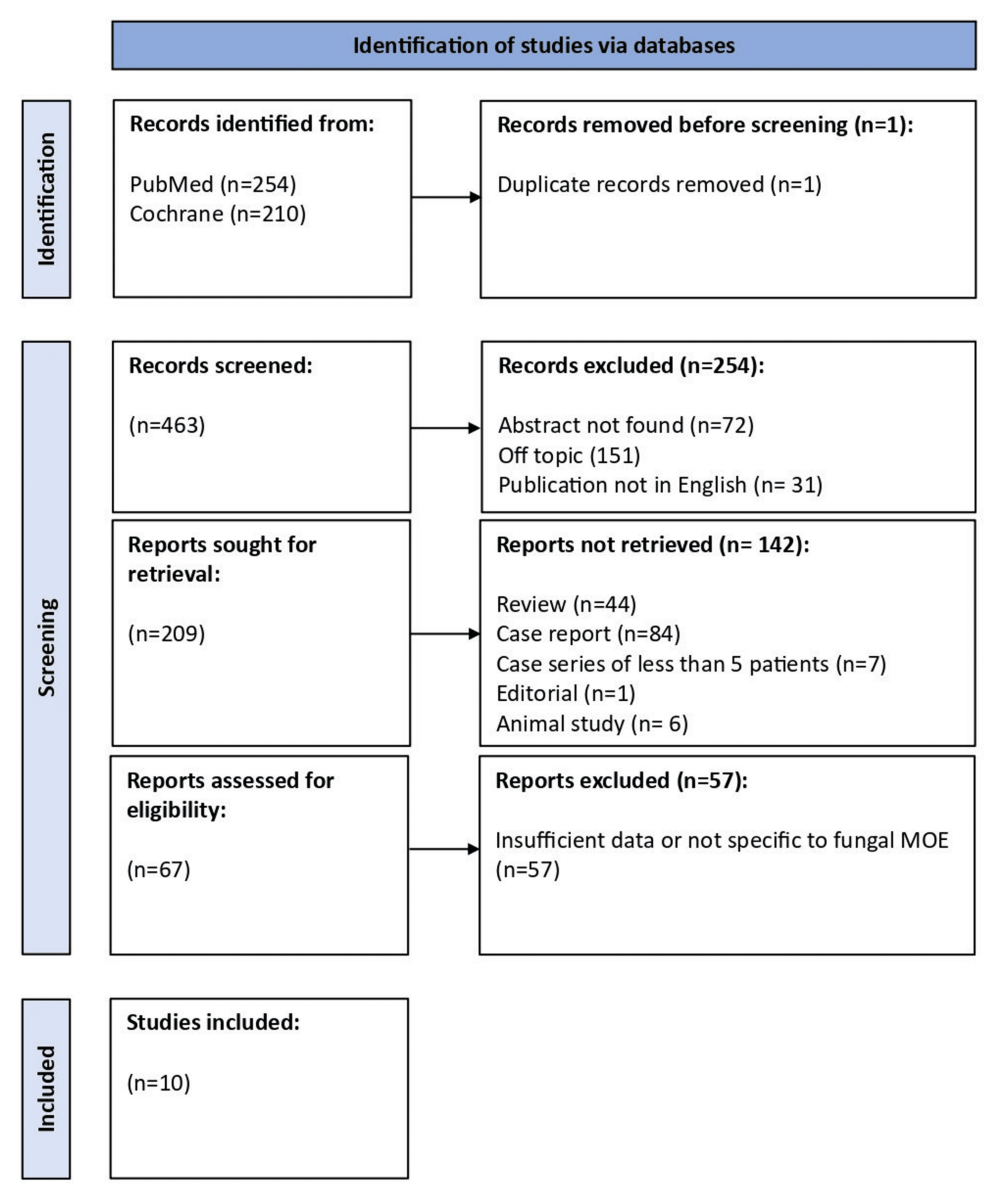
PRISMA flowchart of the search for fungal malignant otitis externa

Data extracted included study characteristics, patient demographics, clinical findings, imaging results, culture findings, treatment, and outcomes. The search strategy and selection process are presented in Figure 1.

Results and discussion

Study and Patient Characteristics

Ten studies were included, encompassing 414 patients, 197 of whom had fungal malignant otitis externa (FMOE) or mixed etiology MOE [10-11,13-20]. Most of the studies were retrospective (nine studies), with only one being prospective. Four studies only included FMOE patients, with the rest describing individuals with MOE of either bacterial or fungal etiology (Table 1). 

**Table 1:** Characteristics of fungal MOE studies included in the present review

Author, Year	Total No. of patients	No. of patients with fungal or mixed etiology MOE
Hasibi et al, 2017 [10]	224	87
Narozny et al, 2006 [11]	8	3
Abu Eta et al, 2018 [13]	52	15
Korbi et al, 2022 [14]	15	15
Hamzany et al, 2011 [15]	9	9
Cho et al, 2020 [16]	34	4
Peled et al, 2020 [17]	10	10
Halwani et al, 2022 [18]	43	41
Marchionni et al, 2016 [19]	12	12
Ress et al, 1997 [20]	7	1
Total	414	197

One hundred and forty-three patients (72.6%) were male, 52 were female (26.4%), and the gender of the remaining two was not specified. The mean age was 65.9 ± 10.6 years. DM was the most common underlying condition, affecting 149 patients (75.6%), while other conditions such as chronic kidney failure, corticosteroid use, chemotherapy, and AIDS were less common. Notably, 42 cases (21.3%) had no documented comorbidities (Table 2).

**Table 2:** Patient profile and demographics

		No. of patients (n=197) (%)
Sex	Male	143 (72)
	Female	52 (26.4)
	N/A	2
Age (years)	Mean ± SD	65.9 ± 10.6
Underlying immunosuppressive conditions	DM	149 (75.6)
	Chronic kidney failure	2 (1)
	Treatment with corticosteroids	2 (1)
	Chemotherapy	1 (0.5)
	AIDS	1 (0.5)
	N/A	42 (21.3)

These findings are consistent with prior studies linking MOE with immunocompromised states, particularly DM [16]. While additional comorbidities, except DM, associated with MOE include hematological diseases and HIV infection, in regards to FMOE, other comorbidities were present in only six patients (3%) [21]. On the other hand, it is not uncommon for MOE to present in otherwise healthy individuals [22]. In addition, based on existing data, fungal MOE shows a predilection for the male gender, with the literature reporting a male-to-female ratio of 2.3 [23]. A similar male-to-female ratio, equal to 2.75, arises from the values in our review.

Clinical Presentation and Imaging Findings

The clinical features of FMOE varied, but headache (93 patients, 47.2%) and otalgia (80 patients, 40.6%) were the most common symptoms, with otorrhea (52 patients, 40.6%) and hearing impairment (33 patients, 6.8%) also being significant. Other symptoms included cranial nerve impairment (54 patients, 27.4%) and temporomandibular joint involvement (14 patients, 7.1%). Imaging showed positive findings in 81 patients (41.1%) through CT or MRI, with bone 99Tc scans positive in 41 patients (20.8%). Gallium scintigraphy (Ga-67) and other imaging techniques were used in 75 cases (38.1%) (Table 3). 

**Table 3:** Clinical and imaging findings

		No. of patients (n=197) (%)
Clinical findings	Otalgia	80 (40.6)
	Otorrhea	52 (26.4)
	Hearing impairment	33 (16.8)
	Edema	1 (0.5)
	Headache	93 (47.2)
	Fever	7 (3.6)
	Temporo-mandibular joint involvement	14 (7.1)
	External auditory meatus stenosis	19 (9.6)
	Granulations	20 (10.2)
	Cranial nerve involvement	54 (27.4)
Positive imaging findings	CT or MRI	81 (41.1)
	99Tc	41 (20.8)
	Ga-67 / Other / N/A	75 (38.1)

Fungal and non-fungal MOE shared several clinical features, including severe otalgia, otorrhea, headache, and hearing loss. However, in our review, headache was the most common presenting symptom, unlike other studies that highlight otalgia as the primary symptom. Taking into account that the profile of MOE has significantly changed since the introduction of the diagnostic criteria by Cohen and Friedman, it is worth revising and broadening our diagnostic approach, paying special attention to patients who seem to deviate from the classical presentation of the disease [6]. This deviation in presentation suggests the need for clinicians to maintain a high index of suspicion in cases that may not present with classic MOE symptoms. Imaging, especially advanced techniques like gallium scintigraphy and 18F-FDG-PET/CT, played an important role in diagnosing and assessing disease progression, with higher sensitivity than standard CT or MRI scans [24-27].

Microbiology

Fungal isolates were identified in 54.3% of the cases, with *Candida spp.* (50 patients, 25.4%) and *Aspergillus spp.* (51 patients, 25.9%) being the most commonly isolated fungi. *Candida albicans* and *Aspergillus flavus* were the most frequent species, with *Aspergillus flavus* appearing more commonly than *Aspergillus fumigatus*, a finding contrary to previous reports. For example, according to Hedayati et al., experimental studies on mice showed that *Aspergillus flavus* could be up to 100 times more virulent as opposed to *Aspergillus fumigatus* and thrives at an optimal growth environment of 37°C, which could potentially justify its stronger pathogenic nature [28]. Other isolated fungi included *Pseudallescheria boydii*, *Malassezia restricta*, and *Geotrichum capitatum*. Fungal MOE often involves mixed infections, either bacterial and fungal or two fungal pathogens (Table 4). 

**Table 4:** Microbiology findings

	Pathogen	No of patients (n=197) (%)
Candida spp.	Total	50 (25.4)
	Candida albicans	23 (11.7)
	Candida tropicalis	6 (3)
	Candida parapsilosis	7 (3.6)
	N/A	14 (7.1)
Aspergillus spp.	Total	51 (25.9)
	Aspergillus fumigatus	11 (5.6)
	Aspergillus flavus	19 (9.6)
	Aspergillus niger	5 (2.5)
	N/A	16 (8.1)
Pseudallescheria boydii		1 (0.5)
Malassezia restricta		1 (0.5)
Geotrichum capitatum		2 (1)
Mixed fungi		2 (1)
N/A / No pathogen detected		90 (45.7)

Treatment and Mortality

Conservative treatment was employed in 179 out of 197 cases (90.8%), with antifungals used in 172 patients (87.3%). Itraconazole was the most commonly administered antifungal, followed by voriconazole. Amphotericin B, previously considered the gold standard, was less commonly used due to its side effect profile. In some cases, patients received a combination of antifungals and antibiotics.

Although, in the past, amphotericin B was the optimal treatment for fungal MOE, voriconazole has now been established as the drug of choice, especially in cases of invasive *aspergillosis*. The same can also be applied to resistant *Candida* infections [15]. Voriconazole can be intravenously administered for long durations, especially in patients with severe comorbidities. The oral form is usually reserved for patients who manifest a better clinical course [8]. However, the resistance rates in both *Aspergillus* and *Candida spp.* often urge its replacement with other antifungals [15]. Depending on the severity of clinical manifestations and occurred complications, a different single or combination of drugs might be considered [8]. Alternative options to voriconazole for invasive aspergillosis include itraconazole and amphotericin B. The relatively limited preference towards amphotericin B could be attributed to its worse side-effects profile and therefore poorer patient tolerance. Special attention needs to be paid to the emerging prevalence of drug-resistant *Candida* and *Aspergillus* forms, which requires the expansion of the available treatment options beyond the field of azoles [29].

Surgical intervention, including local debridement and mastoidectomy, was performed in 35 patients (17.8%), and hyperbaric oxygen therapy was used in 34 of them (17.3%). Surgical interventions included local debridement, canal wall-up mastoidectomy, canal wall-down mastoidectomy, or mastoidectomy with decompression of the facial nerve [17]. Prompted by the reports of the study conducted by Lucente et al., the medical community became more aware that, without prior confinement of the disease using conservative therapeutic methods, surgical treatment as the first-line option may result in adverse outcomes [30]. Moreover, in cases where antifungal and/or antibacterial therapy does not lead to remission of the infection, supplementary treatment methods should be considered, such as hyperbaric oxygen [11].

Mortality in fungal MOE varies between studies, with reported rates ranging from 2% to 11% [14,22]. In our review, the mortality rate was 4% (eight patients), indicating a significant mortality rate despite treatment (Table 5).

**Table 5:** Patient treatment course and outcomes

Treatment			No. (%)
Medication	Antifungal	Fluconazole	31 (15.7%)
		Voriconazole	44 (22.3%)
		Itraconazole	66 (33.5%)
		Amphotericin B	31 (15.7%)
	Combination of antifungal & antibiotic(s)		6 (3.5%)
	Antibiotic(s) only		1 (0.5%)
	N/A		18 (9.1%)
Other	Surgery		35 (17.8%)
	Hyperbaric Oxygen		34 (17.3%)
Course and outcomes	Mean duration of hospitalization (days)		31
	Death		8 (4%)

Late diagnosis, cranial nerve involvement, and inadequate treatment likely contribute to the higher mortality rates seen in some cases. Older age and DM also appear to increase the risk of death in MOE patients, consistent with previous findings [16].

Limitations and Recommendations

This systematic review provides valuable insights into the field of fungal MOE, but several limitations must be considered. The included studies varied in design, sample size, and patient management, which created challenges in data compilation and classification. Additionally, most of the studies were small, single-center reports, limiting the generalizability of the findings. Larger, multicenter studies are needed to draw more definitive conclusions. Moreover, many studies did not clearly specify whether fungal involvement was the primary infection source or a secondary complication, which limits the ability to make precise distinctions about the nature of the disease. Lastly, most of the available studies were case reports, which were not included in this review.

## Conclusions

Fungal MOE, though rare, is a severe condition that primarily affects immunocompromised individuals and requires careful diagnostic consideration alongside non-fungal MOE due to similar clinical presentations. Early inclusion of antifungal treatments and collaboration with specialists may enhance patient outcomes, particularly in cases with poor prognosis. Given the limited data on fungal MOE, expanding research and continuously updating treatment guidelines are essential for optimizing management and ensuring standardized care for affected patients.
